# Effects of *Anethum graveolens L*. on *In Vitro* Matured Mouse Oocytes and Granulosa Cells

**Published:** 2018

**Authors:** Malihezaman Monsefi, Bahareh Khalifeh, Samaneh Nikeghbal

**Affiliations:** Department of Biology, Faculty of Sciences, Shiraz University, Shiraz, Iran

**Keywords:** *Anethum graveolens* L., Culture, Granulosa cells, Oocytes

## Abstract

**Background:**

According to previous studies, *Anethum graveolens* L. (dill) aqueous extracts decreased the fertility of female rats. Therefore, the present study aimed to examine the effects of this herb on cultured granulosa cells and immature oocytes.

**Methods:**

The cells were obtained from 27–29 day immature superovulated mice. The oocytes were cultured in a petri dish consisting of 30 *μl* drops of MEM-α and granulosa cells in a 24-well plate consisting of DMEM/F12 and different concentrations of 0, 10, 50, 100, 500, 1000, 10000 *μg/ml* of dill seed aqueous extract (DSAE) in 37°*C* and 5% CO_2_. Then, the *in vitro* maturation of oocytes, including Germinal Vesicle (GV), Germinal Vesicle Breakdown (GVBD), and meiosis II (MII) and oocyte bioviability were determined. Granulosa cells were then extracted and their bioviability, apoptosis, chromatin condensation, and lipid synthesis were examined. Estrogen and progesterone concentrations and Alkaline Phosphatase (ALP) activity were measured by RIA and spectrophotometry respectively from the supernatant of granulosa cell culture.

**Results:**

The results revealed that concentration of 10000 *μg/ml* of DSAE were toxic and damaged granulosa cell growth and oocytes maturation. Lower concentrations were the same in the control group and did not have any side effects on cell growth. The number of lipid droplets, estrogen and progesterone concentrations, and ALP activity increased with higher doses of DSAE compared to those in the control culture. Additionally, apoptosis and chromatin condensation increased in higher concentrations of DSAE-(500 and 1000 *μg/ml*) treated cells. This herb extract decreased the oocytes maturation in dose-dependent manner.

**Conclusion:**

It was concluded that DSAE increased granulosa cells activity but damaged oocytes maturation, therefore it might be introduced as infertility agent.

## Introduction

Population growth control is of great importance in many countries. Oral contraceptives as steroidal compounds have been used to control fertility. Although these drugs act as potent anti-fertility agents, they are not devoid of side effects such as long-term menstrual cycle irregularities and breast cancer. Hence, the search for natural anti-fertility compounds with minimal side effects is in progress.

*Anethum graveolens* L. (dill), belonging to the umbelliferae family, has been employed to increase milk production and to induce menstruation in Iranian traditional medicine 1. Dill leaf is also used as food flavouring and aroma 2. Iranian people often use dill seed for menstrual cycle regulation and dill leaf for cooking. Therefore, we examined the effects of this herb on the female reproductive system, a topic on which we could not find any scientific reports. Our previous study confirmed a significant lengthening of the estrous cycle and the diestrus phase, and an increase in the progesterone concentration in female rats treated with dill extracts, but the stereological study did not reveal any changes in the volumes of ovaries, and the primary, secondary, and graafian follicles 3. The ultrastructural study of the corpus luteum granulosa cells of these animals revealed an increase in the smooth and rough endoplasmic reticulum and mitochondria, which, in turn, indicated their steroidogenesis 4.

Glucose-6-phosphate and lactate dehydrogenase activities increased in the serum of female rats given the dill seed extract 5. Glucose-6-phosphate activity is increased by estrogen and plays a role in oocyte maturation 6. Lactate dehydrogenase is used as a target enzyme for infertility studies in mammals 7. A significant decrease was observed in the weights and crown rump lengths of foetuses, weights of the placenta, and the fertility index of the dill-seed-treated pregnant rats, while the anti-implantation and anti-fertility indices increased in these animals 5. We examined the four fractions of dill seed extracts such as water, N-butanol, chloroform, and ether fractions to comprise the effects of different components of this herb on sex hormones and duration of pregnancy, as well as the number, weight and crown-rump length of the rat newborns. We concluded that each fraction induced some changes in the mentioned parameters. The use of crude extract in case of infertility was, therefore, recommended 8.

These investigations revealed that dill seed, not dill leaf, might be used as an anti-fertility agent in an animal model by sex hormones elevation. It was important to understand if this herb affected the oocytes or their supporting granulosa cells that are critical for reproduction. Therefore, in the present study, the effects of dill-seed extracts on oocytes In Vitro Maturation (IVM) and granulosa cell culture were examined directly.

## Materials and Methods

### Animal

Immature Balb/c female mice (25–35 days) weighing 13–18 *g* were purchased from the Animal House of Shiraz University of Medical Sciences, Shiraz, Iran. The mice were adapted to the laboratory conditions for two weeks prior to the experiments. Animals were kept at a controlled temperature (22–24*°C*) and a 12 *hr* light-dark cycle (lights on from 6:00 until 18:00); they had free access to food and tap water. The animal experiments were approved by the Institutional Animal Ethics and Health Committee of the Biology Department of Shiraz University, and were performed according to the principles of the care and use of laboratory animals established by the National Institute of Health.

### Preparation of extract

*Anethum graveolens L*. (dill) seeds were prepared from a commercial source in Shiraz (Fars Province, Southern Iran). It identified by a botanist in the Herbarium of Biology department, college of Sciences, Shiraz University, Shiraz, Iran. A voucher specimen was preserved for reference with the serial number 1015. Dill seeds were powdered, and 50 *g* of powder was percolated with 150 *ml* of distilled water for 24 *hr*. Subsequently, the mixture was filtered and concentrated under reduced pressure using a rotary evaporator and vacumed desiccators 9,10. The yield (w/w) of the *Anethum graveolens* L. seed aqueous extract (DSAE) was 6.5 (g/g).

### Ovarian granulosa cells culture

The mice were stimulated by an *i.p*. injection of 7 *IU* Pregnant Mare Serum Gonadotropin (PMSG) (Hypra-Spain). The animals were sacrificed 48 *hr* later by cervical dislocation and the ovaries were removed into DMEM (Dulbecco’s Modified Eagle’s Medium)/F12 (Gibco, USA) supplemented with 20% Fetal Bovine Serum (FBS) (Gibco, USA), 1% penicillin-streptomycin (Sigma-Aldrich, USA) and 0.1% Bovine Serum Albumin (BSA) (Gibco, USA). The pellet containing granulosa cells were aseptically harvested by aspiration from follicles with a 25-gauge needle and released in medium. They were centrifuged (500 *g* for 5 *min*), then resuspended in cultivation medium and seeded. Cells number and viability were estimated using a haemocytometer under a light microscope after vital staining with trypan blue (Sigma-Aldrich, USA). Number of 3×10^5^ cells were grown and maintained in 0.5 *ml* DMEM/F12 containing 20% FBS, and 1% penicillin-streptomycin and 0.1% BSA per each well of 24 well plate. *Anethum graveolens L*. aqueous extract (DSAE) were added to the culture media at concentrations of 0, 10, 50,100, 500, 1000 and 10000 *μg/ml* after 24 *hr*. Osmolality of the extract containing media was adjusted to 300–320 *mosmol* with an osmometer (Gonotec GmbH, Rinteln, Germany). All cells were incubated at 37*°C* with 5% CO_2_ for two days. Each concentration was repeated in 3 wells of well plate and in three 24 well plates separately.

### Cell viability assay

Cell viability was assessed by neutral red (0·05 %) (Merck, Germany) for 2 *hr* at 37*°C*, after which the cells were fixed in formal Ca 1 *min* at RT and washed 2 *min* in saline. Subsequently, 1 *ml* alcohol acid was added and the mixture was incubated 2 *hr* at RT. The optic density of the eluted neutral red in alcohol acid was measured at 540 *nm* wavelength by spectrophotometer (Shimadzu UV-120-01, Kyoto, Japan).

### Estradiol and progesterone measurements

Number of 3×10^5^ cells were grown in 0.5 *ml* DMEM/F12 containing 20% FBS, and 1% penicillin-streptomycin and 0.1% BSA per each well of 24 well plate. Dill seed aqueous extract were added to the culture media at concentrations of 0, 10, 50,100, 500 and 1000 *μg/ml* after 24 *hr*. Granulosa cells were incubated at 37*°C* with 5% CO_2_ for 48 *hr*. Each concentration was repeated in 3 wells of well plate and in three 24 well plates separately. For hormone preservation, the medium were not exchange during this period. Then the supernatant were collected from each well and centrifuged, and then estradiol concentration in different DSAE dosage was measured using Radioimmunoassay (RIA) by estradiol kit (Diasource, distributor Aria Pharmed Producing and Trading, Tehran, Iran) and progesterone concentration were measured using Immunoradiometric Assays (IRMA) by progesterone kit (Diasource, distributor Aria Pharmed Producing and Trading, Tehran, Iran) in Department of Hormonal assay, Research Center of Namazi Hospital, Shiraz, Iran.

### Alkaline phosphatase (ALP) activity assessment

Granulosa cells were cultured with the same method for hormonal measurement. Alkaline phosphatase activities of granulosa cell supernatants were examined by ALP kit (Kimia Pajouhan, Tehran, Iran). ALP converted colorless paranitrophenyl phosphate as a substrate to yellow paranitrophenol and phosphate. Color intensity has a direct proportion with enzyme activity that was measured by spectrophotometer (Jenway, Staffordshire, England) at 405 *nm* wavelength.

### Chromatin condensation assay

Granulosa cells were cultured on 12 *mm* round sterile coverslips that put bottom per each well of 24 well plate and then were treated with different doses of DSAE for 48 *hr*. Granulosa cells were fixed in 3% glutaraldehyde in PBS 0.2 *M* 30 *min* then stained with 5% aniline blue (Acros Organics, USA) in 4% acetic acid 10 *min* at pH=3.5. The coverslips were removed and put on slide and examined by light microscope, and then their photographs were taken by digital camera (Nikon, Japan). Condensed chromatin was stained as dark blue. The light intensity of 100 nuclei in each concentration was analyzed by Image Java software. This software represents light intensity as a number in the range between 0 to 255 which 0 represents the absolute black and 255 represents the absolute white. Cells with more condensed chromatin look darker and acquire lower score in the Image Java calculation.

### Acridine orange/Ethidium bromide (AO/EB) staining

AO/EB staining is used for evaluation of nuclear morphology in apoptotic cells. After the treatment period, granulose cells were harvested and rinsed with PBS. The pellets were resuspended in AO/EB solution including 5 *μl* of AO (Merck, Germany) and 5 *μl* of EB (SinaClon, Iran). After 10 *min*, the cells were put on slide and observed using a fluorescence microscope (Nikon Eclipse-E600) and photographs were taken at ×100 magnification using a digital camera (Nikon, Japan). Acridine orange is a vital dye and stains both live and dead cells. Ethidium bromide stains only cells that have lost membrane integrity. Live cells will appear uniformly green. Early apoptotic cells stain green and contain bright green dots in the nuclei as a consequence of chromatin condensation and nuclear fragmentation. Late apoptotic cells also incorporate ethidium bromide and therefore stain orange, but, in contrast to necrotic cells, the late apoptotic cells show condensed and often fragmented nuclei. Necrotic cells stain orange, but have a nuclear morphology resembling that of viable cells, with no condensed chromatin.

### Oil red o staining of granulosa cells

Granulosa cells secrete steroid hormones, therefore they have some lipid droplets in their cytoplasm. Oil red o is a lysochrome (fat-soluble dye) used for staining of neutral triglycerides and lipids. Granulosa cells were cultured in different DSAE concentrations in 24-well plate. The bottom of each well was covered by sterile round coverslip previously. After 72 *hr*, the culture medium was discarded and cells were fixed with 4% formalin contained 1% calcium chloride for 15 *min* and washed with 70% ethanol. Then 500 *μl* oil red o (Sigma-alderich, USA) solution (250 *mg* oil red o were dissolve in 5 *ml* of 99% isopropanol then 3 parts of this solution were added to 2 parts of dH_2_O) were added to each well. After 15 *min* dye solution was discarded and cells washed with 70% ethanol and dH_2_O respectively. The coverslip were removed and placed on a glass slide and observed by light microscope. Granulosa cells were evaluated as three groups of low, medium and high containing of lipid droplets. Then 100 cells were counted and the percent of each group were recorded in different DSAE concentration.

### Collection of oocytes

Oocytes were obtained from 25–35 days old Blab/C female mice. The mice were stimulated by an *i.p*. injection of 17 *IU* Pregnant Mare Serum Gonadotropin (PMSG) (Hypra, Spain) and 16 *IU* of Human Chronic Gonadotropin (HCG) (LG Life Sciences, South Korea) after 48 *hr*. The animals were sacrificed 16 *hr* later by cervical dislocation and the ovaries were removed into minimum essential medium-alpha (MEM-Alpha) (Gibco, USA) supplemented with 10% FBS (Gibco, USA), 1% penicillin-streptomycin (Sigma-Aldrich, USA).

Oocytes of ovarian follicle were aseptically harvested by aspiration from follicles with a 22 and 31-gauge sterile needles and released in medium under a stereomicroscope. Cumulus cells were removed by repeated pipetting and the oocytes were collected for *in vitro* fertilization. A total of 900 denuded oocytes were obtained from 16 ovaries of 8 mice (each 2 mice in 4 repeated examinations) and they were used for *in vitro* maturation. The average number of collected oocytes was 50 per ovary.

### In vitro maturation (IVM)

The collected oocytes in each examination were randomly divided into control and seven experimental groups. Each group was placed in 35 *μl* micro drops of maturation medium that consisted of MEM-Alpha supplemented with 10% FBS and 1% penicillin-streptomycin over laid with embryo tested light mineral oil and incubated for 24 *hr* in a humidified atmosphere of 5% CO_2_ at 37*°C*. In experimental groups DSAE at concentrations of 5, 10, 50, 100, 250, 500, 1000 and 10000 *μg/ml* were added to the culture media. Osmolality of the extract containing media was adjusted to 300–320 mosmol with an osmometer (Gonotec GmbH, Rinteln, Germany). After 24 *hr* incubation, oocytes were observed by stereomicroscope. Morphological changes in the nucleus or extrusion of condensed oocytes and the first polar body (MII) were used at the criterion for nuclear maturation of Germinal Vesicle (GV) stage oocytes in each DSAE concentration and then their media were exchanged freshly. After 48 *hr*, oocytes in different group were observed again and the percent of each oocyte stages were recorded.

### Oocytes staining

After 48 *hr*, viability of oocytes was estimated after vital staining with trypan blue (Sigma-Aldrich, USA). In live oocytes with intact cell membranes trypan blue was not absorbed therefore it was not colored. However, this dye was traversed the membrane in a dead cell and were showed as a distinctive blue color under a microscope.

The structures of the chromosomes in different stages of oocytes in each concentration were determined by aceto-orcein staining. Oocytes were fixed in an aceto-methanol (acetic acid: methanol, 1:3) solution for 24 *hr* at 4*°C*. Fixed oocytes were transferred onto a microscope slide, and were covered by coverslip. Oocytes were incubated 10 *μl* of aceto-orcein solution (1% orcein, 45% acetic acid) for 2–3 *min* under coverslip and then the structure of the chromosomes was then analyzed.

### Statistical analysis

The data were analyzed with One-Way ANOVA, followed by the Tukey and Scheffe tests. Statistical analyses were done with SPSS version 17.5. The p< 0.05 was considered as statistically significant difference.

## Results

Cell viability assays showed that 10 to 1000 *μg/ml* of DSAE concentrations did not significantly affect granulosa cells, which remained the same as those in control culture. However, at 10000 *μg/ml* of DSAE concentration, the viable granulosa cells decreased significantly 6.3-fold compared to those in the control culture (Table 1).

**Table 1.** The effects of different doses of *Anethum graveolens L*. (dill) seed aqueous extract (DSAE) on cell viability, chromatin condensation and lipid droplets amount of cultured granulosa cells of mice

**DSAE (*μg/ml*)**	**Granulosa cell viability**	**Chromatin condensation**	**Lipid droplets amount**		

			**Low**	**Medium**	**High**
**Control**	0.69±0.19	161.65±2.66	57.57±0.83	20.17±2.9	22.26±1.00
**10**	0.60±0.10	160.75±5.92	33.07±0.93^a^	34.62±0.38^a^	32.31±1.15^a^
**50**	0.57±0.08	158,68±5.21	37.47±1.47^ab^	43.42±0.60^ab^	19.11±0.89^ab^
**100**	0.52±0.22	157.72±9.35	23.65±0.50^adc^	27.23±1.24^adc^	49.11±0.90^adc^
**500**	0.47±0.27	151.53±8.67^c^	15.39±1.00^abcd^	28.85±0.70^abc^	57.76±0.94^abcd^
**1000**	0.43±0.28	148.46±19.41^abcde^	20.58±0.92^abcde^	14.70±0.80^abcde^	64.71±1.00^abcde^
**10000**	0.11±0.03^abcd^	-	-	-	-

a: Significantly different from 0 *μg/ml* (p<0.05);

b: Significantly different from 10 *μg/ml* (p<0.05);

c: Significantly different from 50 *μg/ml* (p<0.05);

d: Significantly different from 100 *μg/ml* (p<0.05);

e: Significantly different from 500 *μg/ml* (p<0.05).

**Figure 1.**
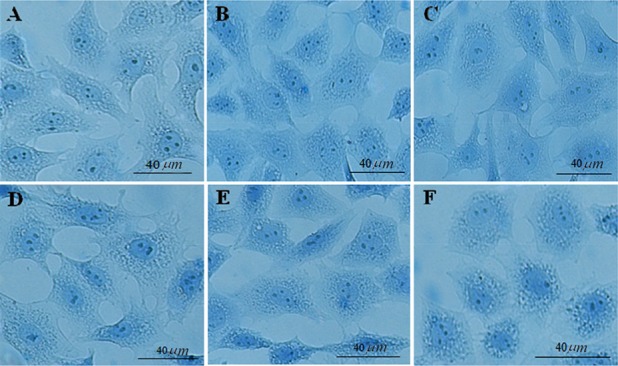
The effects of different doses of *Anethum graveolens L.* (dill) seed aqueous extract on chromatin condensation of cultured granulosa cells, aniline blue staining, scale bar=40 *μm*. A) Control culture, B) 10 *μg/ml*, C) 50 *μg/ml*, D) 100 *μg/ml*, E) 500 *μg/ml* and F) 1000 *μg/ml* of *Anethum graveolens L.* extract treated culture.

Furthermore, the use of aniline blue staining revealed the granulosa cells in the control culture showed the round nuclei of euchromatin in the centre of their cytoplasm. Chromatin condensation of 10, 50 and 100 *μg/ml* DSAE concentrations were nearly the same as control culture. Higher concentrations of DSAE (500 and 1000 *μg/ml*) showed a significantly more condensed chromatin than that of the control granulosa cells (Table 1, Figure 1).

**Figure 2.**
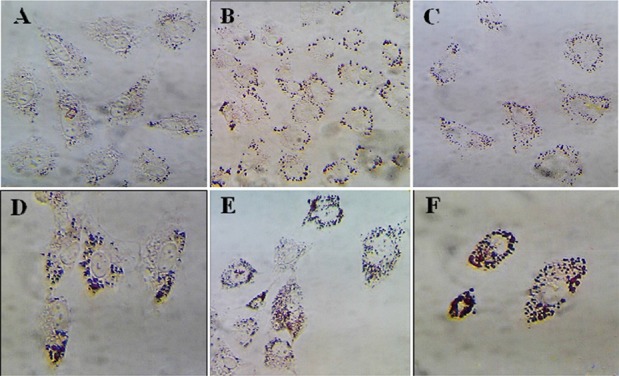
The effects of different doses of *Anethum graveolens L.* (dill) seed aqueous extract on lipid droplets content of cultured granulose cells of mice, oil red staining, ×100 magnification. A) Control culture, B) 10 *μg/ml*, C) 50 *μg/ml*, D) 100 *μg/ml*, E) 500 *μg/ml* and F) 1000 *μg/ml* of *Anethum graveolens L* extract treated culture.

Granulosa cells of the control culture showed the lowest percentage of high lipid droplet cells, low percentage of medium lipid droplet cells, and the highest percentage of low lipid droplet cells. After treatment with different doses of DSAE, granulosa cells showed a significantly higher percentage of high lipid droplets cells but a lower percentage of the medium and low lipid droplet cells compared to the control culture. DSAE concentrations of 500 and 1000 *μg/ml* revealed the highest amount of high lipid droplet cells (Table 1, Figure 2).

**Figure 3.**
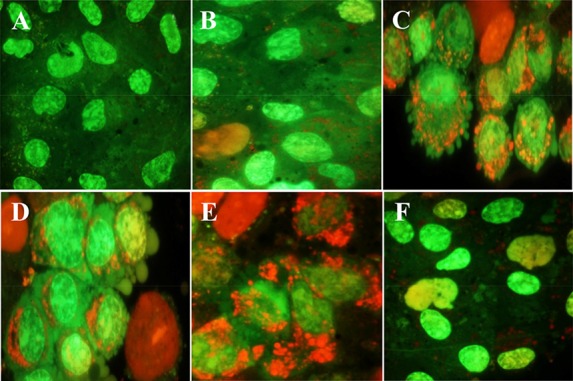
The effects of different doses of *Anethum graveolens L.* (dill) seed aqueous extract on apoptotic activity of cultured granulosa cells, acridine orange/ethidium bromide staining, ×100 magnification. A) Control culture B) 10 *μg/ml*, C) 50 *μg/ml* D) 100 *μg/ml*, E) 500 *μg/ml* and F) 1000 *μg/ml* of *Anethum graveolens L.* extract treated culture. Please note to viable cells with green nucleus, early apoptotic stage cells with light green or yellowish nuclei, chromatin condensation and the formation of blebs on the cell surface and apoptotic nuclei with red nuclei.

Granulosa cells of the control culture (almost 98%) showed nuclei of a dark green colour using AO/EB staining (Figure 3A). These cells of 10 concentration of DSAE were the same as those of the control culture but with light green nuclei (Figure 3B).

Granulosa cells of 50 *μg/ml* of DSAE were similar to 10 *μg/ml* but showed low red punctuate in their cytoplasm and a few cell membrane blebbing (Figure 3C). Granulosa cells in 100 *μg/ml* DSAE concentration cultures showed light green nuclei with more membrane blebbing and cytoplasm granulation than the 50 *μg/ml* cultures. Some dead cells were shown by red-coloured nuclei in this dose treatment culture (Figure 3D). Cells treated with 500 *μg/ml* revealed more red granules in their cytoplasm when compared to 100 *μg/ml* cultures (Figure 3E). The percentage of light green nuclei in 1000 *μg/ml* treated cells increased but the red granules in cytoplasm decreased significantly (Figure 3F). The number of dead cells with red nuclei increased compared to the 500 *μg/ml* treated culture.

Estradiol and progesterone concentrations of the granulosa cells of DSAE-treated cultures increased gradually but were higher in the 500 *μg/ml* DSAE concentration compared to the control cultures (Table 2). However, they showed reverse results in1000 *μg/ml* of DSAE-treated cells, while estradiol and progesterone concentrations decreased compared to the 500 *μg/ml* treated culture (Table 2).

**Table 2.** The effects of different doses of *Anethum graveolens L.* (dill) seed aqueous extract (DSAE) on estradiol and progesterone and alkaline phosphatase activity of cultured granulosa cells of mice

**DSAE (*μg/ml*)**	**Estradiol concentration (*pg/ml*)**	**Progesterone concentration (*ng/ml*)**	**Alkaline phosphatase activity**
**Control**	32.12±13.09	1.02±0.19	2454.30±34.25
**10**	44.00±20.29	1.23±0.73	2456.90±13.88
**50**	45.11±14.29	3.24±1.99	2460.10±19.31
**100**	58.92±16.35	4.00±3.47	2472.20±14.83
**500**	73.68±22.16^ab^	6.43±5.05^ab^	2491.90±15.21
**1000**	68.81±20.23^a^	5.15±2.17	2522.20±24.72^abc^

a: Significantly different from 0 *μg/ml* (p<0.05);

b: Significantly different from 10 *μg/ml* (p<0.05);

c: Significantly different from 50 *μg/ml* (p<0.05).

The ALP activity of granulosa cells in control culture and DSAE concentrations of 5, 10, 50, 100 and 500 *μg/ml* were similar. This enzyme activity signifycantly increased in the 1000 *μg/ml* DSAE concentration compared to the control culture (Table 2).

**Figure 4.**
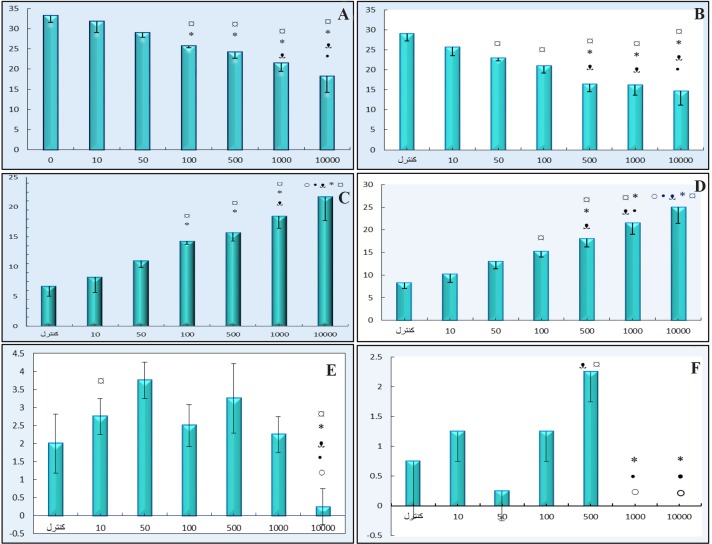
The effects of different doses of *Anethum graveolens L.* (dill) seed aqueous extract on number of different stages of GV (germinal vesicle), GVBD (germinal vesicle break down) and MII (metaphase II) after 24 and 48 *hr* cultures. Number of GV oocytes after 24 *hr* (A) and 48 *hr* (B) cultures, number of condensed oocytes after 24 *hr* (C) and 48 *hr* (D) cultures, number of GVBD oocytes after 48 *hr* (E) and number of MII after 48 *hr* (F) cultures. 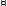
 Significantly different from 0 *μg/ml* (p<0.05); * Significantly different from 10 *μg/ml* (p<0.05); 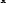 Significantly different from 50 *μg/ml* (p<0.05); • Significantly different from 100 *μg/ml* (p<0.05); ○ Significantly different from 500 *μg/ml* (p<0.05).

Figure 4 represents the number of GV, condensed, GVBD and MII stages of oocytes. The number of GV oocytes decreased significantly in DSAE-treated cultures after 24 and 48 *hr*, but were prominent in higher doses of the extract such as 500, 1000 and 10000 *μg/ml* compared to the control culture. Meanwhile, the oocytes, with condensed cytoplasm and nuclei, increased in treated cultures after 24 and 48 *hr* (Figure 4). The number of GVBD oocytes increased in 500 and 1000 *μg/ml* treated cultures non-significantly but the number of MII oocytes increased in 500 *μg/ml* treated culture compared to the control culture. There were no MII oocytes in1000 *μg/ml* treated cultures. The oocytes stages showed significant differences statistically when compared among different doses as well.

The trypan blue and aceto-orcein staining revealed that the breakdown of nuclear membrane and starting of meiosis II reduced or stopped in a higher dose of DSAE-treated culture (Figure 4).

## Discussion

According to present study results, different doses of DSAE did not show any side effects in granulosa cell viability except for the highest dose of 10000 *μg/ml* concentration that proved toxic, leaving the highest possible number of cells dead. It is important that the other doses, especially higher doses of DSAE such as 500 and 1000 *μg/ml*, did not reveal toxic effects when compared with the control culture. Granulosa cells were mostly viable and their nuclei were euochromatic in appearance even in a 1000 *μg/ml* DSAE concentration and a small population of granulosa cells showed either condensed chromatin after aniline blue staining or apoptotic signs after Ao/Eb staining (Table 1, Figures 1 and 3). Chromantin condensation were referred to compaction and hyperchromic nuclei of granulosa cells before they died. During cell death, chromatin undergoes a phase change from a heterogeneous, genetically active network to an inert, highly condensed form. To put it differently, as DSAE dose increased, granulosa cells showed greater activity in lipid biosynthesis in a way that their cytoplasm was full of with lipid droplets. The high amount of estradiol and progesterone concentrations confirmed increased lipid droplet amounts in a dose-dependent manner.

ALP activity also increased in a higher dose of DSAE-treated culture. ALP is a hydrolase that usually attached to cell membranes, removes the phosphate from organic esters, and facilitates materials movement through the cell membrane. ALP activity was controlled by ovarian hormones 11. Estrogen, along with progesterone, increased the ALP activity and the endometrial thickness 12. The data of *in vitro* evaluation of ovarian granulosa cells confirmed the results of our previous *in vivo* studies as mentioned in the introducetion. We suppose that the reason for our obtained results might be dill phytoestrogens. Dill consists of flavonoids like kaempferol, myristicin, and vicenin. Kaempferol and vicenin showed phytoestrogen properties 13,14. Phytoestrogens are nonsteroidal components that are similar to natural estrogens such as 17β-estradiol. They attach to alpha and beta estrogen receptors and induce biological effects such as cell growth, differentiation, and general homeostasis of reproductive and other systems 15,16.

The similar effects of daidzein, the phytoesrogen of soybean, on pig granulosa cell culture have been reported 17. Most of the phytoestrogens and industrial chemicals behaved as estrogen in the stimulation of ALP activity 18. Oocytes might be classified as an immature or Germinal Vesicle (GV) stage in which its nucleus is in the prophase stage of the first meiosis.

The breakdown of the germinal vesicle (GVBD) indicates a resumption of meiosis and the extrusion of the first polar body, indicating the completion of the first meiotic division and the starting of the second meiosis and stops in its metaphase stage. *Anethum graveolens L*. decreased the oocytes maturation stages in a dose-dependent manner. Oocytes treated with 100 *μg/ml* and higher doses decreased the GV stage and increased the condensed oocytes after 24 *hr*. The rate of decrease in the number of GV oocytes was 1.26-fold in 100 *μg/ml* and 1.5-fold in 1000 *μg/ml* of DSAE concentrations. But almost half the oocytes (in 1000 *μg/ml*) remained in the GV stage and did not reveal cytoplasm and nucleus damage. The same behaviour was observed after 48 *hr* of culture compared to the control culture. Low dose of genistein and diadzein (as phytoestrogens) and their metabolites did not affect oocyte maturation, but their high doses caused oocyte apoptosis 19,20. A low dose of quercetin as an anti-oxidant improved the *in vitro* development of porcine oocytes by decreasing reactive oxygen species levels, but, in higher doses, showed toxic effects and inhibited oocyte growth 21,22. Lower doses of resveratrol protected mouse oocytes from methylglyoxal that induced oxidative damage and inhibited oocytes in the GV stage 23.

## Conclusion

Our data revealed that *Anethum graveolens L*. aqueous extract did not show any harm to granulosa cells even at higher doses (500 and 1000 *μg/ml*). The important point to consider is that these doses (500 and 1000) are too high for *in vitro* studies. The viable granulosa cells, their brief condensation, high hormonal secretion, and ALP activities showed this herb was harmless to the ovary. Therefore, it may be used as a regulatory agent of the reproductive system and for the control of fertility. The effect of this herb on oocytes confirmed its anti-fertility property.
